# The personal and national costs of mental health conditions: impacts on income, taxes, government support payments due to lost labour force participation

**DOI:** 10.1186/1471-244X-11-72

**Published:** 2011-04-28

**Authors:** Deborah J Schofield, Rupendra N Shrestha, Richard Percival, Megan E Passey, Emily J Callander, Simon J Kelly

**Affiliations:** 1 NHMRC Clinical Trials Centre and School of Public Health, University of Sydney, Camperdown, NSW 1450, Australia; 2 NHMRC Clinical Trials Centre, University of Sydney, Camperdown, NSW 1450, Australia; 3 National Centre for Social and Economic Modelling, University of Canberra, Canberra, Australia; 4 University Centre for Rural Health (North Coast), University of Sydney, Lismore, NSW 2480, Australia

## Abstract

**Background:**

Mental health conditions have the ability to interrupt an individual's ability to participate in the labour force, and this can have considerable follow on impacts to both the individual and the state.

**Method:**

Cross-sectional analysis of the base population of Health&WealthMOD, a microsimulation model built on data from the Australian Bureau of Statistics' *Survey of Disability, Ageing and Carers *and STINMOD, an income and savings microsimulation model was used to quantify the personal cost of lost income and the cost to the state from lost income taxation, increased benefits payments and lost GDP as a result of early retirement due to mental health conditions in Australians aged 45-64 in 2009.

**Results:**

Individuals aged 45 to 64 years who have retired early due to depression personally have 73% lower income then their full time employed counterparts and those retired early due to other mental health conditions have 78% lower incomes. The national aggregate cost to government due to early retirement from these conditions equated to $278 million (£152.9 million) in lost income taxation revenue, $407 million (£223.9 million) in additional transfer payments and around $1.7 billion in GDP in 2009 alone.

**Conclusions:**

The costs of mental health conditions to the individuals and the state are considerable. While individuals has to bear the economic costs of lost income in addition to the burden of the conditions itself, the impact on the state is loss of productivity from reduced workforce participation, lost income taxation revenue, and increased government support payments - in addition to direct health care costs.

## Background

Mental health conditions are a highly prevalent condition in numerous countries[1]. Within Australia it is the third largest proportion of the burden of disease[2]. In 2007, almost half of the Australians aged 16 to 84 had experienced a mental health condition at some stage in their lives, and in 2005 the prevalence rate of long-term mental illness was 11% (a figure which has progressively increased since 1995) [3,4].

Due to the high prevalence rates, the economic costs of mental health conditions are large[1]. In Australia in 2004-05, AU$4.1 billion (£2.3 billion) was allocated to be spent on mental health [5]. In the United Kingdom in the same period the £4.5 billion was invested in adult mental health services [6]. In addition to these large national costs, the personal economic costs are also great: people with mental health conditions are recognised as being some of the most socially marginalised and economically disadvantaged members of the community[7].

Mental health conditions have significant impacts on an individual's employment. The labour force participation rates of people with a mental health condition are generally poor - people with a mental health condition have unemployment levels of 75-90% in the US; and 61-73% in the UK. Within Australian individuals with a mental health condition have unemployment rates up to four times higher than healthy Australians[8].

While less prevalent in older age groups [9], older workers who suffer from mental health conditions may be more likely to retire from the workforce early, due to a combination of the effects of ageing and the disabling impacts of the mental health condition[8]. Indeed, within Australia more than half of those with a mental health condition who are aged 45 to 64 years are not in the labour force (106 100 individuals) [10] and this results in reduced savings for these individuals [11].

However, there have been no detailed, comprehensive studies on the economic impacts of early retirement due to mental health conditions to both the individual and the state in Australia. The studies that have looked at the indirect costs of mental illness have generally focused only on loss of income from wages and salaries. They exclude, for example, reduction in income from other sources and for government reductions in income taxation revenue and an increase in social security payments.

This paper quantifies, for the 45 to 64 year old Australian population, the amount of income available to those who have retired early due to depression and also for those with other mental health conditions, the amount of taxation revenue these individuals pay to the Australian government, and the amount of government benefits paid to these individuals. It will quantify the difference of these values between those who have retired early due to depression and other mental health conditions and those in the labour force with no health condition to give a more complete picture of the costs of mental health conditions, and show how much better off the effected individuals and the government would be if the conditions had been prevented and individuals remained in the labour force. It also quantifies the aggregate cost to the state from lost taxation revenue, increased social welfare payments, and estimates the national GDP loss due to individuals exiting the labour force due to depression and other mental health conditions.

## Methods

### Data

The output dataset of a microsimulation model, Health&WealthMOD, which is Australia's first microsimulation model of health and disability, was used to analyse the associated impacts that ill health has on labour force participation, personal income, and government revenue and expenditure. It was specifically designed to measure the economic impacts of ill health on Australian workers aged 45 to 64 years.

The base population of Health&WealthMOD was unit record data extracted from the Survey of Disability, Ageing and Carers conducted by the Australian Bureau of Statistics in 2003[12]. From this dataset, individual records were extracted for those aged 45-64 years. The details extracted for each individual in the base population included demographic variables (for example, age, sex, family type, state of residence, and ethnic background), socioeconomic variables (level and field of education, income, benefits received), labour force variables (labour force participation, employment restrictions, retirement), and health and disability variables (chronic conditions, health status, type and extent of disability, support and care required).

Using a separate microsimulation model--STINMOD--additional economic information such as individual income, government support payments and tax liability was imputed onto the base data. STINMOD is Australia's leading model of income tax and government support payments [13,14] and is maintained and developed for the Australian Government by the National Centre for Social and Economic Modelling. Income and wealth information was imputed onto the base population of Health&WealthMOD by identifying persons with similar characteristics on STINMOD and "donating" their income and wealth information onto

Health&WealthMOD using a process commonly used in microsimulation modelling called 'synthetic matching'[15]. Nine variables: sex (2 groups), income unit type (4 groups), type of government pension/support (3 groups), income quintile (5 groups), age group (4 groups), labour force status (4 groups), hours worked per week (5 groups), highest educational qualification (2 groups) and home ownership (2 groups), that were common to both datasets and strongly related to income were chosen as matching variables for synthetic matching. To check the assumption that the information contained on the STINMOD records accurately reflected the economic status of those records on the 2003 SDAC they were matched with, the accuracy of the matching of these variables were checked. It was found that each of the matching variables on each record were matched within a 5% accuracy, except for age, which was matched within 6% accuracy [16]. Due to the very small matching error it is not expected that the results in this study will be meaningfully affected.

The data were then aged to reflect the 2009 Australian 45 to 64 year old population. The up-rating was used to account for the disability and illness, demographic, labour force, earnings growth and other changes that had occurred between 2003 and 2009. The process by which Health&WealthMOD was built is outlined in detail in Schofield *et al*. [17].

The Survey of Disability, Ageing and Carers provides detailed self-reported data on socio-demographic status, labour force participation and health and disability status (such as chronic conditions) for each individual in Health&WealthMOD. Respondents health conditions were classified by the Australian Bureau of Statistics using ICD10 codes which were developed by the World Health Organisation and endorsed by the 43^rd ^World Health Assembly in 1990 [18]. People who reported their main long term health condition as depression/mood affective disorders (excluding postnatal depression) (ICD code F30-39) were considered to have 'depression'. Those who reported their main long term health condition as mental and behavioural disorders, dementia, schizophrenia, phobic and anxiety disorders, nervous tension/stress attention deficit disorder/hyperactivity, and other mental and behavioural disorders (ICD codes F00-29, F40-99) were categorised as 'other mental health conditions' for this study. Depression was analysed separately from other mental illnesses due to its high prevalence in the population.

In this study those who reported to be out of the labour force due to their illness and listed depression as their main condition were considered to be out of the labour force due to depression. Those who reported being out of the labour force due to their illness and listed one of the other mental health conditions as their main condition were deemed to be out of the labour force due to other mental health conditions. All people who are out of the labour force, regardless of the reason for it, are assumed to be permanently retired.

### Statistical methods

Initial descriptive analysis was undertaken to determine the mean and median weekly income, taxation payments, and social security benefits attributable to individuals employed full time, employed part time, not in the labour force due to depression and and not in the labour force due to other mental health conditions. Income was defined as total gross income from all sources, including employment earnings, transfer income, and income from other sources such as investment properties. A multiple linear regression model of the log of weekly income was used to analyse the differences between weekly incomes of people in the labour force (full-time and part-time) with no health condition and people not in the labour force due to depression and due to other mental health conditions. Analyses were repeated for weekly transfer income and weekly tax liability. Age group, sex and highest education were adjusted for in all regression models. Regression analysis was undertaken on log-transformed data in order to satisfy the assumptions of linear regression analysis, and regression diagnostics confirmed that the assumptions were reasonably satisfied.

The national economic impacts of depression and other mental health conditions, when it leads to exit from the labour force were estimated with the assumption that people who reported being out of the labour due to depression or other mental health conditions would have the same labour force participation rates as of the people with no chronic condition if they did not have the mental health condition. Some of these people who were out of the labour force due to depression and other mental health conditions might still have other chronic conditions other than the mental health conditions (which they cite as their main condition). These other conditions might keep them out of the labour force even if they did not have depression and other mental health conditions. However, there was no data available to estimate what proportion of these people would be out of the labour force due to other chronic conditions if they did not have depression and other mental health conditions. Thus, we conducted a sensitivity analysis assuming:

(1) that if individuals who were out of the labour force due to depression and other mental health conditions did not have these conditions that they would otherwise have had the same labour force participation rates as people with no chronic health conditions, or

(2) that individuals who were out of the labour force due to depression and other mental health conditions would otherwise have had the same labour force participation rates as people with conditions other than depression and other mental health conditions. This assumption was used as the sensitivity analysis for estimating the national economic impacts.

The impact of depression and other mental health conditions on national GDP was calculated based on the Commonwealth Treasury's GDP formula:

where GDP = Gross Domestic Product; H = total hours worked; EMP = total number of persons employed; LF = total labour force; and Pop15+ = population aged 15 years and over [19]

The analyses were undertaken using SAS V9.1 (SAS Institute Inc., Cary, NC, USA). All statistical tests were two sided with the significance level set at 5%. Currency figures are given in 2009 Australia dollars - 1Australian dollar = approximately 0.55GBP in 2009. In 2009 the Purchasing Power Parity (PPP) was 1.46 for Australia and 0.619 for the United Kingdom with the United States being 1. PPP represented the number of monetary units to buy the same representative basket of consumer goods and services [20].

## Results

Amongst those surveyed in the Survey of Disability, Ageing and Carers who were aged between 45 and 64 years, there were 43 individuals who were out of the labour force due to depression, and 54 individuals who were out of the labour force due to other mental health conditions; there were 2 273 who were employed full time with no chronic health condition, and 781 who were employed part time with no chronic health condition. Once weighted, these data represented 25 200 individuals not in the labour force due to depression, 35 200 individuals not in the labour force due to other mental health conditions, 1, 410, 000 individuals employed full time with no chronic health condition, and 421 300 individuals employed part time with no chronic health condition within the Australian population aged 45 to 64 years.

Those who were out of the labour force due to depression had a median weekly income (income from all sources, including government transfer income) of $3671(£202), and those out of the labour force due to other mental health conditions $312 (£172). This is around half of the median weekly income of those employed part-time with no condition ($657 per week (£361), and around one-quarter of the median weekly income of those employed full time with no chronic condition, $1 226 (£ 674) (Table 1).

**Table 1:** Average and median* weekly income, transfer payments and tax liability by labour

Labour Force Status	Weekly income AU$ (£) received by individuals			Weekly transfer income AU$ (£) received by individuals			Weekly tax liability (includes Medicare levy) AU$ (£) paid by individuals		
	
	Mean	SD	Median	Mean	SD	Median	Mean	SD	Median
Employed fulltime, no chronic health condition	1 507 (£829)	33 575	1 226 (£674)	9 (£5)	1 082	0 (£0)	344 (£189)	11 746	223 (£123)

Employed part time, no chronic health condition	657 (£361)	11 714	559 (£307)	28 (£15)	1 661	0 (£0)	78 (£43)	3 066	30 (£17)

Not in labour force due to depression	367 (£202)	6 147	286 (£157)	228 (£125)	3 563	254 (£140)	15 (£16)	1 143	0 (£0)

Not in labour force due to other mental health conditions	312 (£172)	4 705	310 (£171)	274 (£151)	3 952	281 (£155)	0 (£0)	27	0 (£0)

*all results given in 2009 Australian dollars (AU)

Of their total weekly income - those not in the labour force due to depression received a median value of weekly transfer income of $254 (£140) (government support payments) and those not in the labour force due to other mental illnesses received $274 (£151); whereas those in employment receive none (as a median value). Not being in employment and typically with little or no other income, those out of the labour force due to depression and other mental illness paid a median value of zero in tax per week - whereas those employed full-time pay a median value of $223 (£123) per week in tax.

When compared to those with no health condition in full time employment and adjusted for age, sex and education, those out of the labour force due to depression receive 73 per cent less per week on average in total income (Table 2), and those out of the labour force due to other mental health conditions receive 78 per cent less. They also pay almost 100 per cent less per week in taxation, and receive significantly more in government transfer payments.

**Table 2:** Differences in average weekly income, transfer payments and tax liability between labour force status, adjusted for age group, sex and education, for the Australian population aged 45-64 years, 2009

Labour force status	Income			Transfer income			Tax liability (includes Medicare levy)		
	
	% difference	95%CI	p-value	% difference	95%CI	p-value	% difference	95%CI	p-value
Employed full-time, no health condition	*Reference*			*Reference*			*Reference*		

Employed part-time, no health condition	-54.5	(-60.1, -48.1)	<.0001	65.7	(27.6, 115.2)	<.0001	-90.1	(-92.8, -86.4)	<.0001

Not in labour force due to depression	-73.2	(-79.9, -64.3)	<.0001	18 018.6	(8 469.9, 38 606.9)	<.0001	-99.9	(-100.0, -99.7)	<.0001

Not in labour force due to other mental health conditions	-77.8	(-86.2, -64.3)	<.0001	27 862.5	(12 367.2, 62 616.5)	<.0001	-100.0	(-100.0, -99.9)	<.0001

Those employed part-time with no long term health condition also have significantly lower incomes, pay less taxation, and receive more in transfer payments than those employed full time. However the percentage differences between those employed full time and those employed part time, is not as great as those employed full time and those not in the labour force due to mental health conditions (Table 2).

When aggregated, the national impact of depression when it leads to exit from the labour force is $1 billion (£0.55 billion) in lost income, $154 million (£84.7 million) in lost taxation revenue, and an additional $129 million (£71.0 million) in government transfer payments per year. When aggregated, the national impact of labour force exit due to other mental health conditions is $1.5 billion (£0.83 billion) in lost income, $124 million (£68.2 million) in lost taxation revenue, and an additional $278 million (£152.9 million) in government transfer payments per year (Table 3) assuming that otherwise those with mental health conditions would have had the same labour force participation rates as people with no chronic health conditions. The results of the sensitivity analysis show that lost income, tax and additional social security payments would be about 10% lower if it were assumed that if individuals who were out of the labour force due to mental health conditions would otherwise have had the same labour force participation rates as people with conditions other than mental health conditions.

**Table 3:** National annual impact of persons not in the labour force due to depression and other mental illness (adjusted for age, sex and education) for the Australian population aged 45-64 years, 2009

	Lost income $AU (£)	Additional transfer payments $AU (£)	Lost taxation revenue $AU (£)
Not in the labour force due to depression	1,018,918,000 (£560,404,900)	129,455,000 (£71,200,250)	154,033,000 (£84,718,150)

Not in the labour force due to other mental health conditions	1,530,353,000 (£841,694,150)	277,655,000 (£152,710,250)	124,055,000 (£68,230,250)

Note: Based on the differences between persons not in the labour force due to depression and other mental illness and the weighted average of persons employed full time and part time with no chronic conditions.

As a result of the 25 200 workers missing from the labour force due to early retirement as a result of depression, there is a annual loss of $698 million (£383.9 million) in GDP. As a results of the 35 200 workers missing from the labour force due to early retirement as a result of other mental health conditions, there is a annual loss of $975 million (£536.3 million) in GDP.

## Discussion

The costs of depression and other mental health conditions are considerable both at the individual level and at the aggregate national level. Individuals aged 45 to 64 years who have retired early due to depression personally have 73% lower income then their full time employed, healthy counterparts and those retired early due to other mental health conditions have 78% lower incomes. This equated to an annual national loss of income of $1 billion (£0.55 billion) for those with depression and $1.5 billion (£0.83 billion) for those with other mental health conditions. The national aggregate impact of depression and other mental health conditions through the loss of labour force participation amongst 45 to 64 year olds, equated to $278 million (£152.9 million) in lost income taxation revenue, $407 million (£223.9 million) in additional transfer payments and around $1.7 billion in GDP in 2009 alone.

A limitation of this study is that the results are based on a relatively small sample size of individuals who are not in the labour force due to depression and other mental ill health - 43 and 54 individuals from the original 2003 SDAC survey respectively. The findings are also based upon cross sectional data from the original 2003 SDAC, rather than longitudinal data, although respondents do identify the reason they left the labour force including whether this was due to illness and what their main health condition was. The findings are also based upon respondents' self-reported data, and as such the potential for bias in the results cannot be excluded. However, self-report health and economic status are regarded as valid measures [21,22].

The direct health costs of treating mental health conditions was estimated to be $4.1 billion (£2.3 billion) for all age groups in 2003-04. This estimate covered health expenditure in hospitals, non-hospital medical services, pharmaceuticals, research, and community mental health services; with the majority being spent on hospital patients and community mental health services[5]. (The United Kingdom, with a population about three times that of Australia invests £3.9 billion per annum in mental health services for adults alone). However, it should be noted that only 62% of those with a mental illness seek medical help in Australia [23] and thus the potential direct medical costs may be higher if adequate services were available. It is also estimated that $1.2 billion (£0.66 billion) is spent on aged care programs in Australia, and significant other amounts on housing and accommodation programs, workforce participation programs and disability services for those people with a mental illness [24].

So while the direct costs are significant, so too are the indirect costs, with the combined costs of lost income, lost taxation revenue, increased government social security payments, and lost GDP in 2009 totalling more than the estimated government expenditure on mental health in 2003. Other studies have estimated the costs of workforce participation in terms of the number of working days lost due to mental ill health, lost income or disability support payments [1,24-32]. However, these studies were more limited in scope and did not include taxation and GDP costs. They also have a number of additional limitations, including only using average earnings, or average disability support payments, to estimate costs of lost income, or only presenting the aggregate national cost - not the cost to individuals [1,28-32].

Average estimates of earnings and disability support payments may not be representative of the population with mental health conditions. Our study used individual level income, tax payment and government support payment data to estimate the cost to individuals because of their early retirement due to mental illness.

There are numerous cost effective drugs for treating mental illness [33-36], these may be used to help overcome the costs to both individuals and governments that can result when conditions impact on the functional capacity of individuals, and lead to early retirement. The UK Department of Health has support the prevention and early treatment of mental health conditions in recognition of the potential to avoid the large financial burden of the disease on the state [37]. However, within Australia only 62% of those with a mental illness seek medical help [23] and as such there is much room for improving the management of these conditions. Amongst those who do seek treatment in Australia, only six visits per year to a psychologist are funded under Medicare [38] as such, there may be gap in what is provided to patients and what is actually required to meet their medical needs, Furthermore, it has been noted that there is a shortage of psychiatrists in Australia [39] that may be leaving some mental health patients without access to care or with long waiting periods. The role for government in supporting the wider uptake of the management or prevention of mental health conditions, can well be justified when the savings in terms of increased labour force participation and the associated avoidance of taxation revenue loss and increased disability payments outlined in this paper are considered.

Despite the increases of government spending on mental health services [40,38], the costs of mental health conditions which falls to governments is still far larger than their spending on mental health services. These arguments provide further support to the need for governments to invest in mental health services and prevention and support measures, to steam these costs[23]. There is currently limited government spending on prevention and early intervention[41,39]. Governments would benefit from prevention and reduction of mental health through increased taxation (income tax, payroll tax, etc), reduced transfer payments, and reduced expenditure on other services (medical, justice, housing, etc)[41,39]. For example, it has been estimated that in Victoria (Australia's second most populated state) a 1% reduction in the burden of mental health would cost around $26 million (AU) and would deliver a net benefit of $7 million (AU)[41,39].

Australia has a poor record of employing those with any disability, ranking amongst the lowest for OECD countries[42,40]. The current Australian employment system is failing to maximise the employment of those with a mental health condition in the labour force[42,40]. This suggests that a multifaceted strategy is required that aims to prevent the onset on mental health conditions, assist sufferers in manage much of their mental health conditions when it is occurring, and also helping individuals remain integrated within society.

## Conclusion

While the cost to government is considerable, the economic cost of mental health conditions to individuals is also large. Due to low rates of labour force participation in their working years, people who suffer from mental health conditions may be more prone to poverty in retirement, due to lack of accumulated savings[8,11]. As such, mental illness can lead to a lifetime of social and economic marginalisation[8].

Furthermore, not only is employment essential for economic security, employment is vital for those with a mental health condition in maintaining a connection with the community, and potentially also for their own mental health (indeed, long-term unemployment itself is associated with mental illness)[42,40]. Employment is important for self-esteem, creating a social identity and places people within social networks [43,41].

## Competing interests

The authors declare that they have no competing interests.

## Authors' contributions

DS led the study and conceived the original study design. RS created the dataset, Health&WealthMOD, and led the generation of the results. RP, MP and SK all contributed expert advice and/or technical assistance in the respective areas of income, mental health and wealth. EC contributed to the generation of results, and drafted the manuscript. All authors contributed to the interpretation of the results, and edited the final manuscript.

## Pre-publication history

The pre-publication history for this paper can be accessed here:

http://www.biomedcentral.com/1471-244X/11/72/prepub
